# Associations between flexibility, stretching habits, and spine injuries in professional golfers: a nationwide, cross-sectional study

**DOI:** 10.1186/s13018-025-06268-z

**Published:** 2025-09-26

**Authors:** Hong Jin Kim, Hyung Rae Lee, Seung Woo Suh, Dong-Gune Chang, Jae Hyuk Yang

**Affiliations:** 1 Department of Orthopaedic Surgery, Kyung-in Regional Military Manpower Administration, Suwon, Korea; 2 Department of Orthopedic Surgery, Korea University Anam Hospital, Korea University College of Medicine, 73 Goryeodae-ro Seongbuk-gu, Seoul, 02841 Republic of Korea; 3 Department of Orthopaedic Surgery, Korea University Guro Hospital, Seoul, Korea; 4 Department of Orthopaedic Surgery, Inje University Sanggye Paik Hospital, Seoul, Korea

**Keywords:** Spine injury, Professional golfers, Flexibility, Stretching

## Abstract

**Background:**

Although pre-training stretching and the inherent flexibility of an athlete are generally regarded as beneficial for injury prevention, limited evidence exists on the association between flexibility, stretching habits, and spine injuries in professional golfers. This study aimed to evaluate the association between flexibility and spinal injuries among professional golfers in South Korea.

**Methods:**

A nationwide cross-sectional study was conducted to assess spine injuries among professional active golfers in South Korea. Data on cervical (C)-spine and thoracolumbar/lumbar (T/L)-spine injury experiences, pre-injury golf-related habits, and flexibility measures containing the floor touch test, shoulder reach test, and thumb-to-forearm test were collected. Post-injury modifications in stretching, warming-up, and swing motion were also assessed. Multivariate logistic regression analysis was used to analyze the risks of golf-related spinal injuries.

**Results:**

Of the 439 participants, 33.7% reported the experience of spinal injuries, with 15.5% experiencing C-spine injuries and 21.9% reporting T/L-spine injuries. For the flexibility measures, the shoulder reach test showed significant differentiation between injured and non-injured golfers (*P* = 0.010), with a “fair” grade being associated with a lower risk of T/L-spine injuries (OR, 0.29; 95% CI, 0.14–0.62). However, the floor touch test did not show a significant distinction (*P* = 0.274). Among golfers with the experience of T/L-spine injury, performance was significantly associated with modifications in warming-up (*P* = 0.002) and swing motion (*P* = 0.033).

**Conclusion:**

This study found that a fair grade of flexibility in the shoulder reach test was associated with a significantly lower risk of T/L-spine injuries. Additionally, modifications in warming-up and swing motion after T/L-spine injuries were significantly associated with the performance of professional golfers.

## Introduction

Among golf-related musculoskeletal injuries, the lower back is the most commonly affected site, primarily due to overuse, which can potentially lead to chronic musculoskeletal issues [1, 2]. Although golf is a non-contact sport, overuse injuries may occur due to improper swing mechanics, particularly at the point of ball impact [3]. The modern golf swing is designed to maximize the hip-shoulder separation angle, resulting in increased shoulder rotation while restricting hip turn [4]. This mechanism can impose excessive stress on the spine through torsional loading from supra-maximal trunk rotation and exaggerated hyperextension during the follow-through [4]. In other words, spinal injuries in golf are understood to occur when excessive strain is placed on the viscoelastic structures beyond their physiological flexibility [3, 5].

Due to the repetitive nature of the swing motion, musculoskeletal injuries in golfers most commonly involve the spine (20–50%), followed by the hand/wrist and shoulder, with spinal injuries accounting for the highest proportion [6]. In a study by Robinson et al., involving 910 amateur golfers, lumbosacral injuries were reported to have the highest incidence and injury burden [6]. As spinal injuries are primarily attributed to overuse mechanisms, degenerative conditions and sprains/strains have been reported as the most frequent types of spinal disorders [3, 7].

To reduce spinal loading, a multidisciplinary golf rehabilitation program recommends three key strategies for patients with golf-related spine injuries: modifying the golf swing motion, enhancing trunk muscle conditioning, and incorporating flexibility exercises [8]. Regarding flexibility, Vad et al. reported that a limited range of motion (ROM) in the hip joint was significantly correlated with increased lumbar spine extension in golfers with a history of low back pain [9]. Meanwhile, although stretching is generally regarded as beneficial for injury prevention by improving ROM and flexibility, a definitive consensus on its effectiveness is yet to be established [10].

Given the limited evidence on the association between flexibility, stretching habits, and spine injuries in professional golfers, we conducted a nationwide, survey-based cross-sectional study to assess the relationship between flexibility, stretching, and spinal injuries in professional golfers. Therefore, this study aimed to investigate the association between flexibility and related golf habits, such as warming-up and stretching, and spine injuries among professional Korean golfers affiliated with the Korean Professional Golfers’ Association (KPGA) and the Korean Ladies Professional Golfers’ Association (KLPGA). Furthermore, among golfers with a history of spinal injuries, we sought to analyze whether habitual changes in stretching, warming-up, and swing motion influenced their golf performance.

## Methods

After approval of the study protocol in our institutional review board (IRB, number: 2024AN0336), this nationwide, survey-based, and cross-sectional study was conducted in accordance with the current guidelines of Strengthening the Reporting of Observational Studies in Epidemiology (STROBE) [11].

### Study design and recruitment of participants

**Fig. 1:**
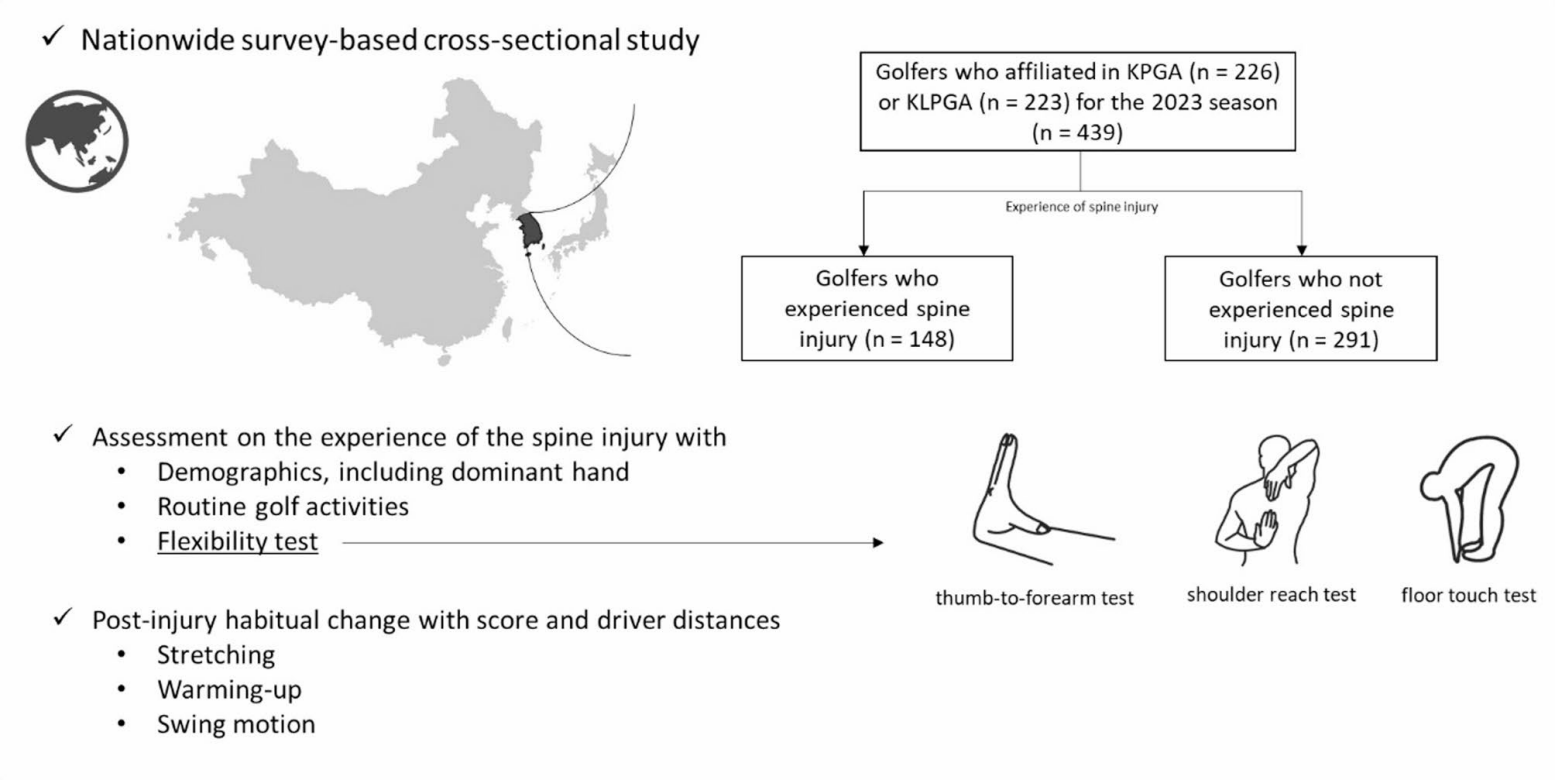
A study scheme including the flow chart and measured flexibility

We recruited active professional golfers registered with the KPGA and KLPGA for the 2023 season through direct communication channels within these organizations. Informed consent was obtained from all participants before participation in the survey. Golfers who did not consent to the survey were excluded from the study. Therefore, the inclusion criteria for this study were being active professional golfers registered with the KPGA or KLPGA during the 2023 season, while the exclusion criteria were not-consent to participate in the study. Consequently, a total of 439 participants–226 from the KPGA and 213 from the KLPGA–participated in this cross-sectional study, achieving a 100% response rate through head-to-head matched interviews for the survey, which included both closed-ended questions for quantitative data and open-ended questions for qualitative insights. The flexibility test was performed by members (sports medicine surgeons) of the Korean Academy of Golf Medicine. We conducted face-to-face surveys and flexibility tests during the 2023 season competitions, and the collected data were recorded in an online-based system. The overview of the study scheme is presented in Fig. 1.

### Data collection and outcome measures

We collected data on the experience of spine injury (including cervical (C)-spine injury and thoracic and/or lumbar (T/L)-spine injury), which encompassed degenerative disease, ligamentous or muscular injuries, and bony injuries. For the experience of spine injury, we asked participants whether they had experienced spinal injuries. We further inquired whether they had visited a hospital due to low back and/or cervical pain and what types of treatment they had received, such as observation, conservative treatment, injection, or surgery. The baseline characteristics included as sex (male or female), dominant hand (left or right), age, height, weight, body mass index (BMI), and golf routines (pro-career years, average rounds per week, and the frequency of golf practice). The three representative tests for the measurement of flexibility were measured, consisting of the floor touch test (no touch, fingertip touched, fist touched, or palm touched), shoulder reach test (fingertips ≥ 5 cm apart [poor grade], fingertips < 5 cm apart [fair grade], fingertip touched [good grade], interlaced finger [very good grade], or touched palm [excellent grade]), and thumb-to-forearm test (fingertips ≥ 5 cm apart from forearm [poor grade], fingertips < 5 cm apart from forearm [fair grade], or touched forearm [good grade]) [12–14]. Regarding the flexibility-related habits, we collected warming-up hours before rounds and practice, and frequency of golf-related stretching exercises, respectively. For the included participants who experienced spinal injuries, we investigated whether there were any changes in scores (improved, no change, or worsened) and driver distance (increased, no change, or decreased) after recovery. Additionally, we examined whether there were any modifications in golf-related habits following the injury (yes/no questions), including enhanced stretching routines (duration and frequency), extended warming-up time, and alterations in the swing motion.

In this study, the primary outcome was to assess the association between flexibility, measured using the floor touch test, the shoulder reach test, and the thumb-to-forearm test, and spinal injuries (C-spine and T/L-spine injuries) among professional golfers in South Korea. The secondary outcome was to investigate the association between post-injury changes in golf performance and habitual modifications (stretching, warming-up, and swing motion) among participants who had experienced spinal injuries.

### Statistical analysis

We conducted statistical analyses using Python (version 3.11.5., Python Software Foundation, Wilmington, DE, USA) with matplotlib (version 3.7.2.). Descriptive statistics were presented as mean values and standard deviations (SD) for continuous variables and numbers and percentages for categorical variables. A normal distribution was confirmed by the Kolmogorov–Smirnov test. After confirming data homogeneity or heteroscedasticity, Student’s *t-*test (independent) was used for continuous variables, and the chi-squared test was used for categorical data, as appropriate. Multivariate logistic regression analyses were performed to identify the association between flexibility, measured using the floor touch test, the shoulder reach test, and the thumb-to-forearm test, and spinal injuries (C-spine and T/L-spine injuries), presenting the odd ratios (ORs) and corresponding 95% confidence intervals (CIs). Statistical significance was set at *P* < 0.05.

## Results

### Baseline characteristics

Among the total of 439 professional golfers, 33.7% (*n* = 148) had experienced spinal injuries. Specifically, C-spine injuries were reported by 15.5% (*n* = 68) of the participants, while T/L-spine injuries were experienced by 21.9% (*n* = 96). A total of 3.6% (16 of 439) of professional golfers experienced both C- and T/L-spine injuries. Golfers with a dominant left hand were significantly more prevalent in the spinal injury group (9.5%, 14 of 148) than in the non-spinal injury group (4.1%, 12 of 291) (*P* = 0.025). There were no statistically significant differences in sex, age, height, weight, BMI, pro-career years, average rounds per week, and frequency of golf practice between the two groups (all *P*s > 0.05) (Table 1).

**Table 1:** The baseline characteristics of professional golf players

Variables	Participants (*n* = 439)	Spine injury (*n* = 148)	No spine injury (n = 291)	*P*
Sex (n, %)				0.870
Male	291 (66.3%)	77 (52.0%)	149 (51.2%)	
Female	148 (33.7%)	71 (48.0%)	142 (48.8%)	
Dominant hand (n, %)				**0.025**
Right	413 (94.1%)	134 (90.5%)	279 (95.9%)	
Left	26 (5.9%)	14 (9.5%)	12 (4.1%)	
Age (n, %)				0.249
≤ 20 years	55 (12.5%)	15 (10.1%)	40 (13.7%)	
21 to 30 years	300 (68.3%)	97 (65.5%)	203 (69.8%)	
31 to 40 years	80 (18.2%)	35 (23.6%)	45 (15.5%)	
41 to 50 years	3 (0.7%)	1 (0.7%)	2 (0.7%)	
> 50 years	1 (0.3%)	0	1 (0.3%)	
Height (cm, mean ± SD)	172.0 ± 7.9	172.3 ± 7.9	171.9 ± 7.9	0.624
Weight (kg, mean ± SD)	72.0 ± 12.0	72.1 ± 11.5	72.0 ± 12.4	0.883
BMI (kg/m^2^, mean ± SD)	24.2 ± 2.8	24.2 ± 2.5	24.2 ± 2.9	0.888
Pro-career years (n, %)				0.641
1 to 3 years	75 (17.1%)	21 (14.2%)	54 (18.6%)	
3 to 5 years	91 (20.7%)	33 (22.3%)	58 (19.9%)	
5 to 10 years	80 (18.2%)	30 (20.3%)	50 (17.2%)	
10 to 20 years	128 (29.2%)	40 (27.0%)	88 (30.2%)	
≥ 20 years	65 (14.8%)	24 (16.2%)	41 (14.1%)	
Average rounds per week (n, %)				0.745
< 1	34 (7.7%)	12 (8.1%)	22 (7.6%)	
1 to 2	141 (32.1%)	52 (35.1%)	89 (30.6%)	
3 to 4	149 (33.9%)	46 (31.1%)	103 (35.4%)	
≥ 5	115 (26.3%)	38 (25.7%)	77 (26.5%)	
Frequency of golf practice (n, %)				0.203
< 1 per week	1 (0.3%)	1 (0.7%)	0	
1 to 2 per week	11 (2.4%)	3 (2.0%)	8 (2.7%)	
≥ 3 per week	49 (11.2%)	15 (10.1%)	34 (11.7%)	
< 2 hours in every day	111 (25.3%)	30 (20.3%)	81 (27.8%)	
≥ 2 hours in every day	267 (60.8%)	99 (66.9%)	168 (57.7%)	

N, number; SD, standard deviation

### Flexibility test and related golf habits

The floor touch flexibility test showed no significant difference between the spine injury and no spine injury groups (*P* = 0.274). However, the shoulder reach flexibility test revealed a significant difference between the spine injury and no spine injury groups (*P* = 0.010), with 23.0% vs. 17.5% in the poor grade, 14.0% vs. 22.0% in the fair grade, 40.5% vs. 41.6% in the good grade, 23.6% vs. 16.8% in the very good grade, and 3.4% vs. 2.1% in the excellent grade for each group. Similarly, the thumb-to-forearm flexibility test showed a significant association (*P* = 0.044), with a higher proportion of fair flexibility in no spine injury group. Regarding pre-injury golf habits related to flexibility, there were no statistical differences in warming-up hours or the frequency of golf-related stretching exercises (all *P*s > 0.05) (Table 2).

**Table 2:** Flexibility, and its-related golf habits in professional golf players

Variables	Participants (*n* = 439)	Spine injury (*n* = 148)	No spine injury (n = 291)	*P*
*Flexibility*				
Floor touch flexibility test (n, %)				0.274
No touch, poor	48 (10.9%)	19 (12.8%)	29 (10.0%)	
Fingertip touched, fair	181 (41.2%)	53 (35.8%)	128 (44.0%)	
Fist touched, good	61 (14.1%)	19 (12.8%)	42 (14.4%)	
Palm touched, excellent	149 (33.9%)	57 (38.5%)	92 (31.6%)	
Shoulder reach flexibility test (n, %)				**0.010**
Fingertips ≥ 5cm apart, poor	85 (19.4%)	34 (23.0%)	51 (17.5%)	
Fingertips < 5cm apart, fair	78 (17.8%)	14 (9.5%)	64 (22.0%)	
Fingertip touched, good	181 (41.2%)	60 (40.5%)	121 (41.6%)	
Interlaced finger, very good	84 (19.1%)	35 (23.6%)	49 (16.8%)	
Touched palm, excellent	11 (2.5%)	5 (3.4%)	6 (2.1%)	
Thumb-to-forearm flexibility test (n, %)				**0.044**
Fingertips ≥ 5cm apart, poor	224 (51.0%)	87 (58.8%)	137 (47.1%)	
Fingertips < 5cm apart, fair	158 (36.0%)	42 (28.4%)	116 (39.9%)	
The forearm touched, good	57 (13.0%)	19 (12.4%)	38 (13.1%)	
*Related golf habits*				
Warming-up hours before rounding (n, %)				0.298
< 30 min	180 (41.0%)	67 (45.3%)	113 (38.8%)	
30 min to 1 hour	194 (44.2%)	58 (39.2%)	136 (46.7%)	
1 to 2 hours	56 (12.8%)	20 (13.5%)	36 (12.4%)	
≥ 2 hours	9 (2.0%)	3 (2.0%)	6 (2.1%)	
Warming-up hours before practice (n, %)				0.287
5 min	61 (13.9%)	16 (10.8%)	45 (15.5%)	
5 min to 10 min	157 (35.8%)	56 (37.8%)	101 (34.7%)	
10 to 30 min	167 (38.0%)	60 (40.5%)	107 (36.8%)	
≥ 30 min	54 (12.3%)	16 (10.8%)	38 (13.1%)	
Frequency of golf-related stretching exercises (n, %)				0.241
< 3 per week	192 (43.7%)	65 (43.9%)	127 (43.6%)	
≥ 3 per week	141 (32.1%)	54 (36.5%)	87 (29.9%)	
< 2 hours in every day	93 (21.2%)	27 (18.2%)	66 (22.7%)	
≥ 2 hours in every day	13 (3.0%)	2 (1.4%)	11 (3.8%)	

N, number

### Multivariate logistic regression analyses for spine injuries

Multivariate logistic regression analysis identified the dominant hand, the shoulder reach flexibility test, and the thumb-to-forearm flexibility test as significant factors associated with the occurrence of spinal injuries. Left-hand dominance (OR, 2.57; 95% CI, 1.10–6.02; *P* = 0.029) was associated with a significantly increased risk of spinal injuries compared to being right-handed. A fair grade on the shoulder reach flexibility test (OR, 0.29; 95% CI, 0.14–0.62; *P* = 0.001) was significantly associated with a decreased risk of spinal injuries compared to a poor grade. Similarly, a fair grade on the thumb-to-forearm flexibility test (OR, 0.58; 95% CI, 0.37–0.93; *P* = 0.024) significantly reduced the risk of spinal injuries compared to a poor grade. No statistically significant differences were observed for other grades in the shoulder reach and thumb-to-forearm flexibility tests.

Specifically, age between 31 and 40 years (OR, 3.70; 95% CI, 1.18–11.63; *P* = 0.025) was a significant factor associated with an increased risk of C-spine injuries. Meanwhile, two significant factors were associated with the risk of T/L-spine injuries: left-hand dominance (OR, 3.46; 95% CI, 1.48–8.08; *P* = 0.004) and a fair grade on the shoulder reach flexibility test (OR, 0.34; 95% CI, 0.14–0.84; *P* = 0.020) (Table 3).

**Table 3:** Multivariate logistic regression analysis for spine injuries

Variables	B	SE	OR (95% CI)	*P*
*Spine injuries*				
Dominant hand				
Right	Ref			
Left	0.95	0.43	2.57 (1.10–6.02)	**0.029**
Shoulder reach flexibility test				
Fingertips ≥ 5cm apart, poor	Ref			
Fingertips < 5cm apart, fair	-1.24	0.38	0.29 (0.14–0.62)	**0.001**
Fingertip touched, good	-0.24	0.28	0.78 (0.45–1.37)	0.392
Interlace to finger, very good	0.27	0.32	1.31 (0.68–2.51)	0.424
Touched to palm, excellent	0.15	0.67	1.16 (0.31–4.32)	0.822
Thumb-to-forearm flexibility test				
Fingertips ≥ 5cm apart, poor	Ref			
Fingertips < 5cm apart, fair	-0.54	0.24	0.58 (0.37–0.93)	**0.024**
Forearm touched, good	-0.31	0.33	0.74 (0.39–1.40)	0.348
*C-spine injuries*				
Age (n)				
≤ 20 years	Ref			
21 to 30 years	0.81	0.54	2.25 (0.78–6.53)	0.136
31 to 40 years	1.31	0.58	3.70 (1.18–11.63)	**0.025**
> 40 years	1.45	1.27	4.25 (0.36–50.82)	0.253
*T/L-spine injuries*				
Dominant hand				
Right	Ref			
Left	1.24	0.43	3.46 (1.48–8.08)	**0.004**
Shoulder reach flexibility test				
Fingertips ≥ 5cm apart, poor	Ref			
Fingertips < 5cm apart, fair	-1.08	0.46	0.34 (0.14–0.84)	**0.020**
Fingertip touched, good	-0.07	0.32	0.93 (0.50–1.73)	0.828
Interlace to finger, very good	0.36	0.35	1.43 (0.72–2.87)	0.308
Touched to palm, excellent	0.16	0.74	1.17 (0.28–4.94)	0.833

N, number; B, unstandardized beta coefficient; SE, standard error; OR, odd ratio; CI, confidence interval; C-spine, cervical spine; T/L-spine, thoracic/lumbar spine

### Post-injury changes in golf performance and flexibility-related habits

**Fig. 2:**
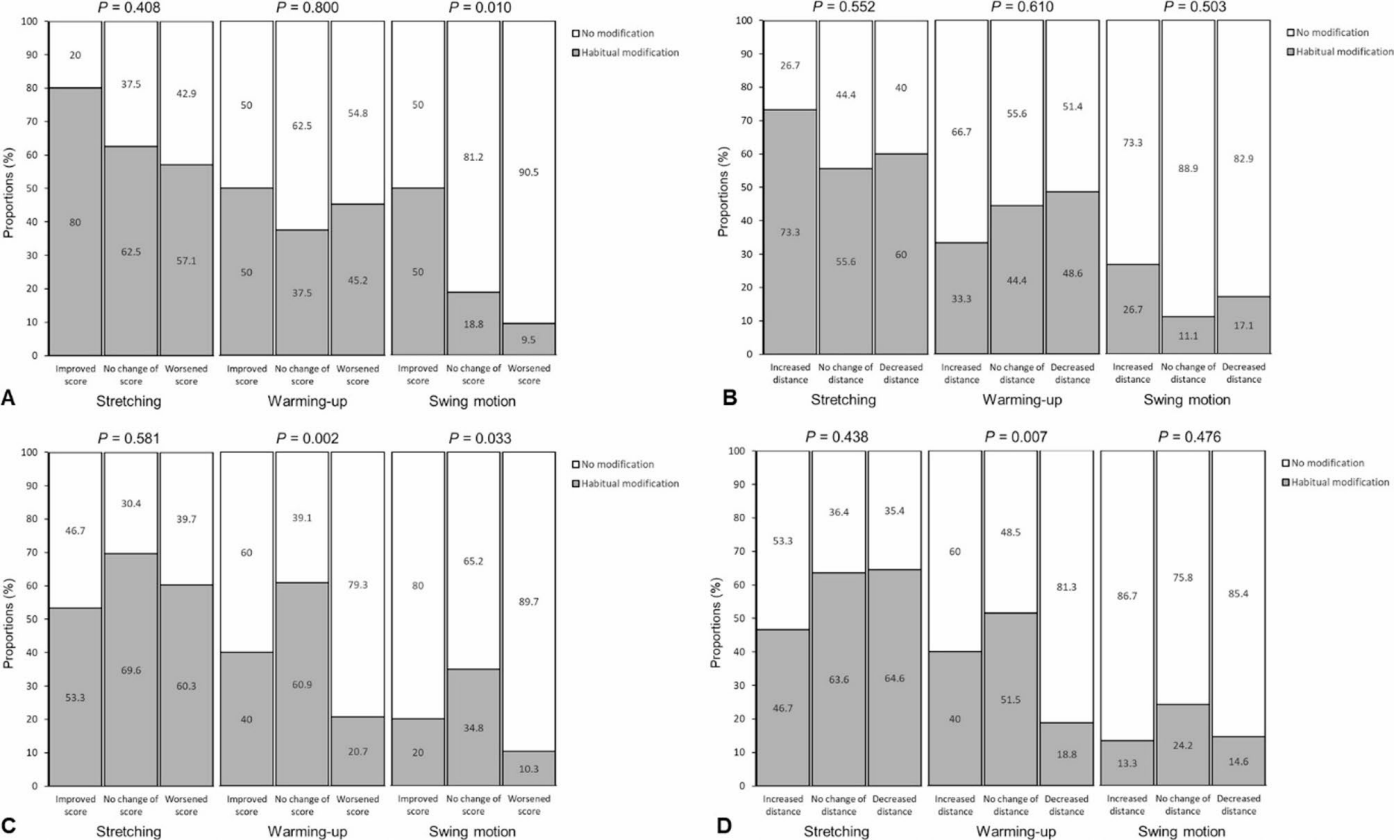
Post-injury golf-related habitual changes. (**a**) Score change after C-spine injury. (**b**) Driver distance change after C-spine injury. (**c**) Score change after T/L-spine injury. (**d**) Driver distance change after T/L-spine injury

We further investigated the association between golf performance (score change and driver distance change) and post-injury golf-related habitual changes (stretching, warming-up, and swing motion). Golf performance was categorized as improved, no change, or worsened, while post-injury habitual changes were assessed as either no modification or modification, based on yes/no questions. Among participants who experienced C-spine injuries, golf score was significantly associated with modifications in swing motion (*P* = 0.010), with 50% of those who improved their score implementing swing modifications, compared to 18.8% of those with no change in score and 9.5% of those whose scores worsened (Fig. 2a). The change in driver distance showed no statistically significant differences in relation to habitual modifications in stretching, warming-up, or swing motion (Fig. 2b). Among participants who experienced T/L-spine injuries, golf score was significantly associated with extended warming-up time (*P* = 0.002) and alterations in swing motion (*P* = 0.033) (Fig. 2c). Driver distance was significantly associated only with extended warming-up time (*P* = 0.007) (Fig. 2d). Specifically, extended warming-up time and modifications in swing motion following spinal injuries effectively prevented declines in golf performance, indicating that scores and driver distances did not worsen.

## Discussion

In our comprehensive cross-sectional study, we investigated the risk factors associated with spinal injuries and their association with stretching-related habitual changes in post-injury golf performance among professional golfers during the 2023 season in South Korea. Our findings demonstrated that flexibility was a significant factor influencing the risk of spinal injuries, particularly T/L-spine injuries. Furthermore, our results on flexibility indicated that a fair grade of flexibility, as measured by the shoulder reach test, significantly reduced the risk of T/L spine injuries. However, no statistically significant reduction in spinal injury risk was observed for good, very good, or excellent flexibility grades compared to the poor flexibility grade. Regarding habitual changes after spinal injuries, modifications in stretching routines did not lead to improvements in golf performance, including score and driver distance. However, improvements in warming-up habits helped prevent declines in performance, as evidenced by the stabilization of scores (not worsened scores) and maintenance of driver distances (not decreased distances), suggesting the preventive role of warming-up exercises in golf performance. Additionally, alterations in swing motion not only improved golf scores following C-spine injuries but also prevented performance deterioration following T/L-spine injuries.

Among the three flexibility tests assessed, the floor touch flexibility test showed no significant association with the presence of spinal injuries. While this test primarily measures back flexion, golf swings are more closely related to axial rotation and lateral flexion [4]. In contrast, the shoulder reach and the thumb-to-forearm flexibility tests demonstrated a significant association, particularly in the fair flexibility grade. These tests are also relevant to golf mechanics, as they assess shoulder rotation and wrist flexibility, both of which are integral to golf swing performance [2, 15]. These findings suggest that a flexibility assessment model may be necessary to better elucidate the relationship between flexibility at golf-specific sites (such as the hip joints) and the risk of injuries [9].

Our study suggested that adequate flexibility (a fair grade) provided a protective effect against spinal injuries among Korean professional golfers. However, excessive flexibility, particularly in the shoulder reach test, did not significantly reduce the risk of such injuries. Excessive flexibility or joint hypermobility may also increase the risk of sports injuries [16, 17]. Hypermobility, characterized by movement beyond the normal physiological range, can lead to joint instability due to laxity of the surrounding ligaments and soft tissues [17]. This instability, when combined with repetitive loading from golf swings, may predispose individuals to overuse injuries. Furthermore, decreased joint stability can place greater demands on periarticular muscles and ligaments, potentially leading to fatigue and an elevated risk of injury [18]. In such cases, injury prevention should emphasize stabilizing exercises focused on muscle strengthening, along with adequate rest to manage fatigue [18]. In line with our findings, we emphasize the importance of individualized injury prevention strategies based on each golfer’s flexibility profile.

The modern golf swing emphasizes a large shoulder turn with a restricted hip turn to maximize swing power, ensure proper sequencing, and maintain consistent ball striking [8]. However, this swing mechanism can lead to supra-maximal trunk rotation, which places excessive strain on the spine [4]. Considering the swing mechanics employed by professional golfers actively competing in the 2023 season, our multivariate logistic regression analysis indicated that hyper-flexibility in the shoulder reach test may be associated with supra-maximal trunk rotation, potentially increasing the risk of overuse injuries [19].

When flexibility is significantly limited, excessive golf swing motion may develop to compensate for the desired posture, potentially increasing the risk of overuse injuries, just as observed in our study when excessive flexibility was present [3]. Previous studies have reported that limited flexibility increases the risk of golf-related injuries [9]. This process can be explained by three mechanisms: excessive stress caused by an insufficient range of motion during the golf swing, a resulting inefficient swing pattern due to compensatory movements, and increased vulnerability to overuse injuries [4]. Specifically, repetitive rotational forces and impact loading can impose excessive strain on the lumbar spine. Such biomechanical stress, driven by compensatory movement patterns, increases the burden on the lumbar musculature and facet joints, thereby predisposing individuals to fatigue and overuse-related spinal injuries—one of the most common forms of injury among golfers. Taken together, the incorporation of structured flexibility training—including stretching—and individualized conditioning programs should be emphasized for golfers with reduced flexibility, as these strategies may play a critical role in both injury prevention and performance enhancement [20].

**Fig. 3:**
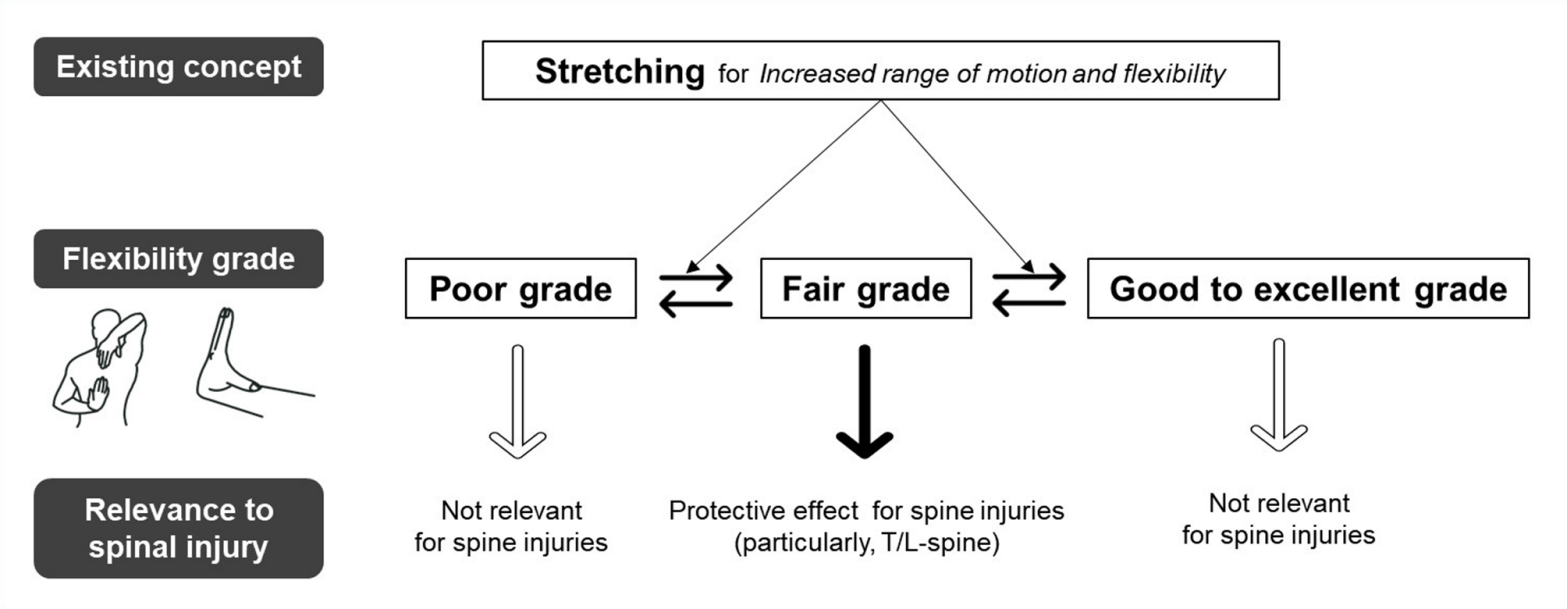
Summary of the results in our study, indicating the limited role of stretching in the prevention of spinal injuries among professional golf players

Although stretching activities are known to improve ROM and flexibility, our findings suggest that stretching plays only a limited role in preventing T/L-spine injuries through increased flexibility (Fig. 3) [21–23]. Therefore, our study, which specifically examined professional golfers, underscores the importance of maintaining an optimal level of shoulder flexibility, adopting a golf posture that minimizes spinal strain post-injury (such as the classic golf swing), and prioritizing comprehensive warm-up routines [8].

These findings on flexibility highlight the importance of transitioning from a modern to a classic golf swing to mitigate the risk of spinal injuries. Moreover, our study suggested that swing modification was potentially associated with the golf scores among actively competing professional golfers. The modern golf swing, characterized by a maximized hip-shoulder separation angle, leads to exaggerated hyperextension and increased stress on the intervertebral discs and facet joints [3]. In contrast, the classic golf swing allows for sufficient hip rotation with a heel raise, which helps reduce spinal stress. Vad et al. supported this hypothesis by highlighting the role of limited ROM in the hip and lower back. In other words, to reduce lower back pain or prevent T/L-spine injuries, it is crucial to modify swing mechanics, as suggested by our study, or enhance hip flexibility to sustain the modern golf swing [9]. However, since we did not account for hip flexibility and specific mechanics of swing motion changes in our analysis, future research is needed to further explore their role in spinal injury prevention.

Trunk muscle conditioning has been emphasized for the prevention of golf injuries [4, 24]. Electromyographic studies have shown that golfers with lower back pain exhibit reduced activity in the erector spinae muscles during the backswing and/or at impact, which may impair the efficiency of the swing motion [25, 26]. Carnovale et al. reported that paraspinal injuries associated with golf were more prevalent than neurological injuries, with muscular strain being the most common injury type (accounting for nearly 58%) [27]. Despite the impact of trunk muscles in golf injuries, our study did not include measures related to paraspinal muscle function, which may act as a potential confounding factor. In the future, further studies considering trunk muscle function will be required to validate and strengthen our findings.

Gosheger et al. reported that engaging in a warm-up routine for more than 10 min before golf activities may be beneficial in preventing injuries and overuse syndromes [23]. Their recommendations for an effective warm-up include exercises such as jumping jacks to increase body temperature, stretching, and simple swing practice [23]. It has been suggested that stretching prior to golf activities serves as a preparatory step within the broader warm-up process. Similarly, Alfonso et al. identified warm-up routines, including stretching, as a critical component of injury prevention, particularly emphasizing their necessity for sustaining long-term sports participation among older individuals [10]. This necessity is attributed to the natural decline in flexibility and the increased prevalence of degenerative joint diseases associated with aging [10, 19]. Our study on professional golfers in South Korea found that while improved warm-up routines stabilized scores and maintained driver distances, stretching alone was not significantly associated with performance among golfers who had experienced T/L-spine injuries. These findings suggest that a comprehensive warm-up approach, rather than isolated stretching, may be more effective for injury prevention and performance optimization.

Regarding additional factors associated with spinal injuries, our study found that left-handed dominance significantly increased the risk of T/L-spine injuries, while age between 31 and 40 years was significantly associated with a higher risk of C-spine injuries. These variables, unlike flexibility, represent non-modifiable factors that are difficult to alter or adjust [28, 29]. For the dominant hand, we suggest that the imbalance between left-handedness and swing direction may have contributed to this increased risk, including the limited availability of left-handed equipment, asymmetrical mechanics between left- and right-handed golfers, and the right-handed dominant training system in South Korea. Most golf training regimens are designed for right-handed players in South Korea, requiring left-handed golfers to adapt to techniques that may not align with their natural biomechanics, thereby increasing their risk of overuse injuries. Since training methodologies and practice drills are predominantly based on right-handed mechanics, left-handed golfers may experience greater physical strain, further exacerbating their susceptibility to injury. However, further investigation is needed to establish a causal relationship in future research.

Our study offers key insights into the relationship between flexibility and golf-related spinal injuries. First, we present data on spinal injuries among professional golfers, an area with limited research. Although golf rehabilitation models emphasize healthy swing mechanics, their effectiveness in preventing back pain remains unclear. Our findings reinforce the importance of swing modifications for injury prevention and performance enhancement. Second, we address the ongoing debate on stretching in sports, identifying a fair flexibility threshold—particularly in the shoulder—that offers a protective effect against lower back injuries [10, 30, 31]. Third, our study aligns with current golf rehabilitation strategies, including swing modifications, trunk muscle conditioning, and flexibility exercises. However, we further emphasize the necessity of a comprehensive approach that integrates both warm-up routines and stretching, particularly for professional golfers. Overall, our findings underscore the need for personalized approach, rather than one-size-fits-all strategy, approach to spinal injury prevention and post-injury performance optimization in professional golfers.

This study has several limitations. First, the limitations of a survey-based analysis, such as recall and response bias, were a potential concern. The injury data relied on self-reported recall and did not capture details such as frequency of injury episodes, thereby introducing a considerable risk of recall bias. Furthermore, our data on the experience of spinal injury did not distinguish specific components of spinal injuries. However, we tried to mitigate these issues by maximizing the survey response rate and ensuring no missing data during the survey process through face-to-face interview. Second, our nationwide survey did not encompass the entire population of professional golfers who actively competed during the 2023 season in South Korea. With a participation rate of 10.3%, there is a limitation in determining whether this sample accurately represents the overall population. To justify the generalizability of our findings, future studies with sufficient statistical power are warranted. Third, the participants in our study were Korean professional golfers, which does not represent the broader ethnic population. While this study focused on the specific population (Korean professional golfers), we did not consider the validity of flexibility measures and survey questionnaires. The questionnaire was comprehensively designed by considering golf-related injuries in Korean professional golfers, but needs to be standardized and validated. Further studies are required using standardized assessment tools, such as methods for recording and reporting of epidemiological data on injury and illness in sports [1]. Last, due to the inherent limitations of a cross-sectional design, our study does not establish a causal relationship between flexibility and spinal injuries. Therefore, future research employing longitudinal designs is warranted to clarify the causal inference underlying this association. Nevertheless, our findings provide valuable insights into the association between flexibility and spinal injuries among active professional golfers, particularly within the Asian athlete population.

## Conclusions

This study found that a fair grade of flexibility in the shoulder reach test was associated with a significantly lower risk of T/L-spine injuries. Additionally, modifications in warming-up and swing motion after T/L-spine injuries were significantly associated with the performance of professional golfers.

## Data Availability

Data are available upon reasonable request due to ethical issues for sensitive personal information.

## Abbreviations

C: Cervical
CI: Confidence interval
IRB: Institutional review board
KLPGA: Korean Ladies Professional Golfers’ Association
KPGA: Korean Professional Golfers’ Association
OR: Odd ratio
ROM: Range of motion
SD: Standard deviations
STROBE: Strengthening the Reporting of Observational Studies in Epidemiology
T/L: Thoracolumbar/Lumbar
